# Skull Base Osteomyelitis With Facial and Trigeminal Nerve Involvement Secondary to Otitis Media

**DOI:** 10.7759/cureus.13394

**Published:** 2021-02-17

**Authors:** Abdulaziz Abuabat, Tariq Tatwani

**Affiliations:** 1 Family Medicine, College of Medicine, King Saud Bin Abdulaziz University for Health Sciences, Riyadh, SAU; 2 Otolaryngology, Prince Sultan Military Medical City, Riyadh, SAU

**Keywords:** skull base osteomyelitis, otitis media, facial nerve, trigeminal nerve

## Abstract

Skull base osteomyelitis (SBO) is a rare condition that is associated with high morbidity rates. It is most commonly caused by an infection in the external part of the ear that eventually spreads to the skull base and related structures. To our knowledge, reports in the literature concerning atypical causes of SBO (e.g., otitis media) have been scarce. In this report, we present a rare case of an elderly diabetic patient who presented with unilateral SBO originating from an infection in the middle ear. This patient also had ipsilateral facial weakness and trismus, indicating a spread of the infection to the facial and trigeminal cranial nerves.

## Introduction

Skull base osteomyelitis (SBO) is a rare condition that arises from an infection spreading from the adjacent tissues, most commonly in the form of malignant or necrotizing otitis externa [1]. Several bones are typically involved in the inflammation in SBO, including the temporal and sphenoid bones. Therefore, this condition is associated with a poor prognosis and high rates of mortality and morbidity [1-2]. Patients with SBO can potentially present with symptoms of cranial nerve involvement since the passage of some of these cranial nerves is in proximity to the skull base [1-2]. Patients with such involvement can present with symptoms such as facial weakness, vocal cord paralysis, and trismus, depending on the cranial nerve involved [1-2]. In this report, we present a case of an elderly diabetic patient who developed SBO secondary to a middle ear infection involving the facial and trigeminal cranial nerves.

## Case presentation

A 65-year-old male patient, who was known to have diabetes mellitus and hypertension, presented to our clinic with a one-month history of right ear pain and discharge. Initially, the patient had begun to experience mild ear pain and discomfort. This pain had worsened with time, and discharge had occurred subsequently. The patient had sought medical treatment at a local hospital in his area in Southern Saudi Arabia. He had been prescribed antibiotics, but they had not improved his condition. Soon after, the patient had developed right-side facial weakness and trismus, which had prompted him to seek treatment at our institution. Upon presentation, the patient reported no fever and no history of recent travel, trauma, or recurrent ear infections. While the patient did have a history of animal contact, he had no history of raw milk ingestion. In addition, the patient had had contact with a pulmonary tuberculosis patient approximately 15 years prior, but he had no reported history of weight loss, night sweats, or loss of appetite.

Upon physical examination, the patient had a visible unilateral mass in the right side of the nasopharynx seen by nasal endoscopy. Otherwise, the nose was clear with no polyps or discharge. However, otalgia and otorrhea were present in the right ear. The perforated tympanic membrane in the right ear was seen by an otoscope. Notable enlargement of the level II lymph nodes was observed on the right side of the neck. The pharynx was clear, and the patient had stable vital signs.

No significant findings were detected in the blood tests. The patient’s complete blood count (CBC) was normal, with no evidence of leukocytosis, anemia, or thrombocytopenia (i.e., all CBC measurements were within normal ranges). The blood chemistry tests were also normal. However, the patient’s C-reactive protein level was markedly elevated at 47 mg/L. A sputum sample was collected for tuberculosis testing, including an acid-fast bacillus (AFB) test and a polymerase chain reaction (PCR), both of which came back negative for tuberculosis.

**Figure 1:**
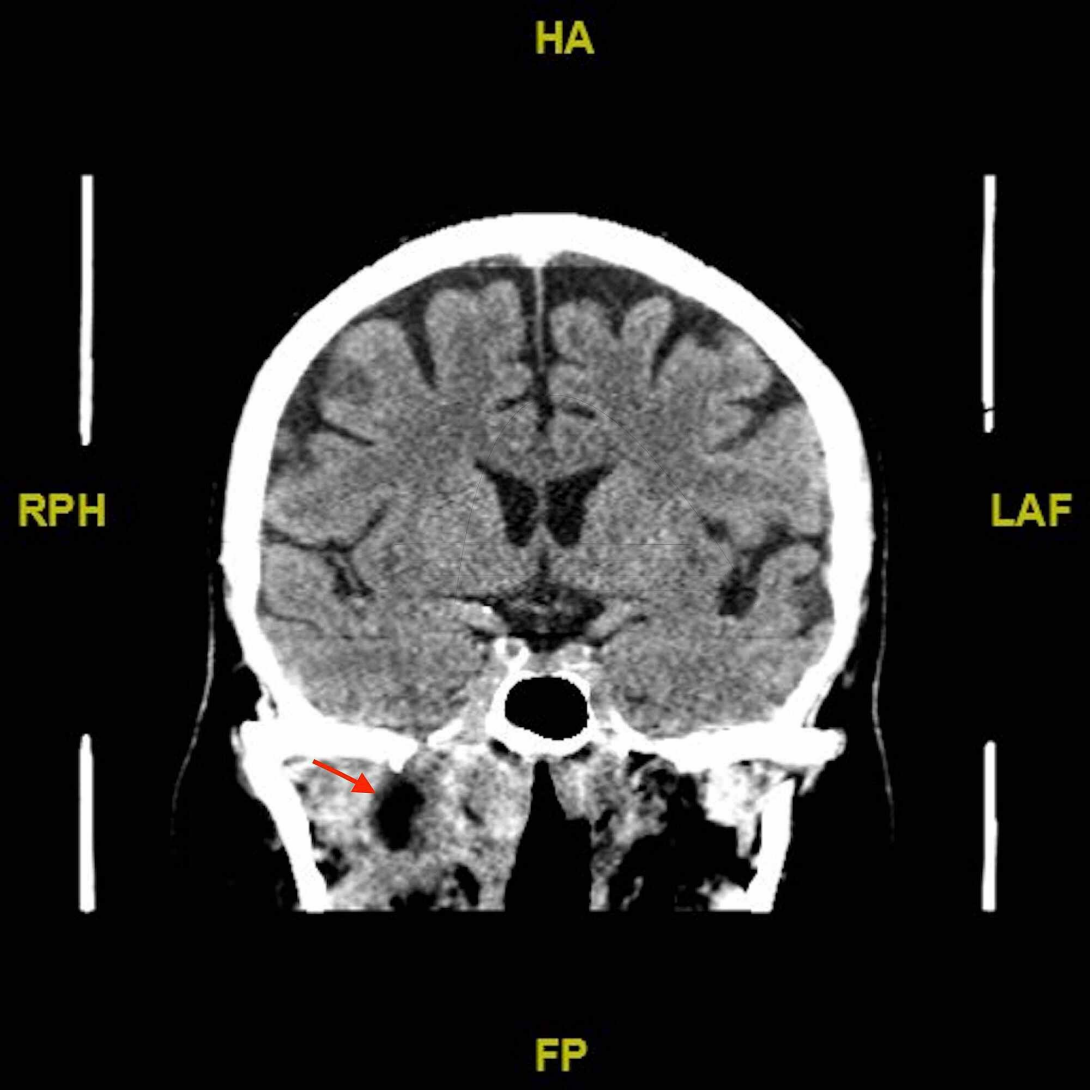
CT scan demonstrating the lesion at the time of presentation CT: computed tomography

**Figure 2:**
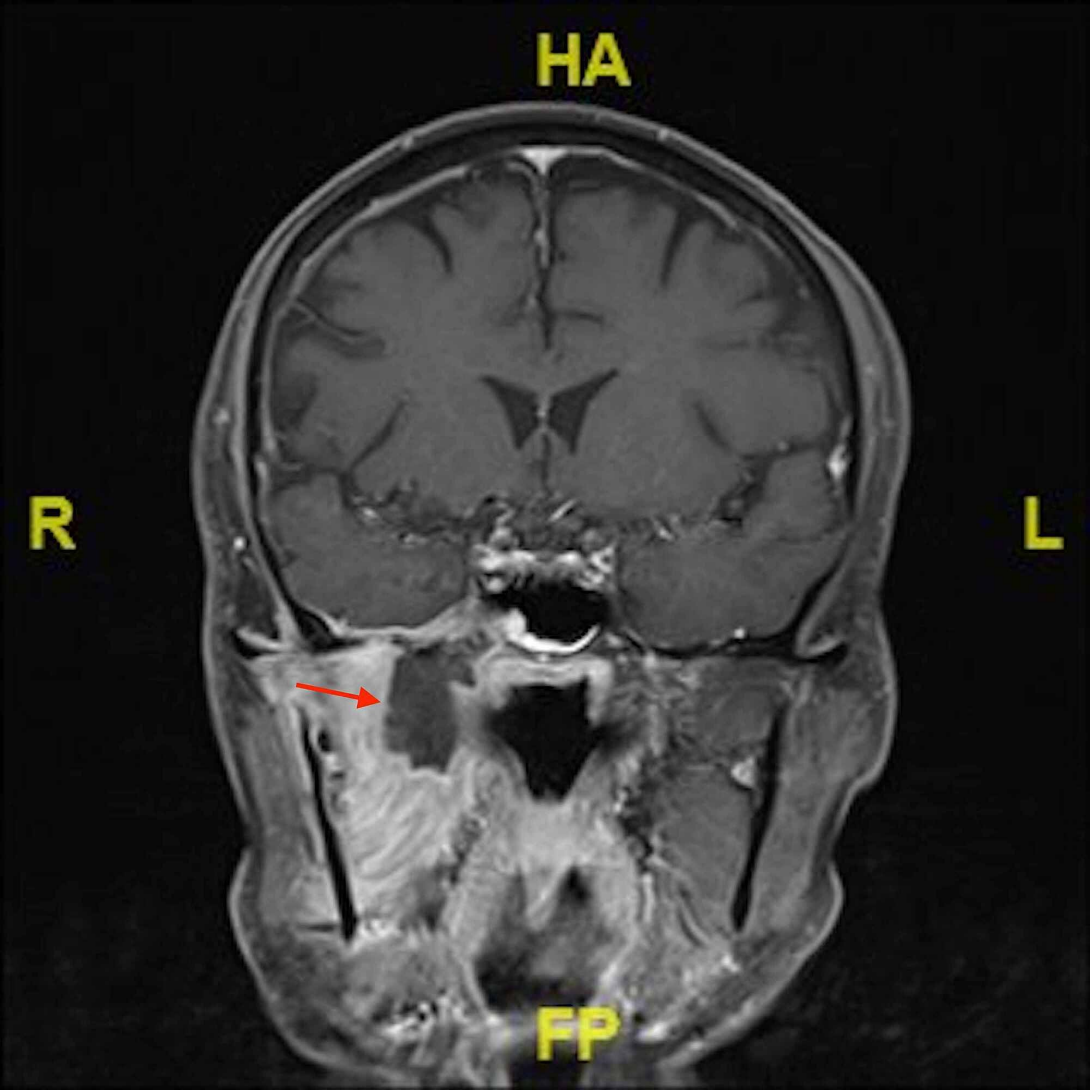
MRI scan demonstrating the hypointense lesion at the time of presentation as seen in T2 (transverse relaxation time) MRI: magnetic resonance imaging

Following the first clinical encounter, the patient was admitted for further investigation of the discovered nasal mass. Initially, a CT scan was requested. The CT scan showed an ill-defined lesion at the right parapharyngeal space extending to the lateral wall of the nasopharynx, pterygoid muscles, and ipsilateral skull base (Figure 1). Radiology recommended MRI for better visualization. The MRI report revealed a significant inflammatory process representing an infection of the right temporal fossa with the central collection, most likely an abscess, and involvement of the right trigeminal nerve (Figure 2). Next, the patient was due for a biopsy to rule out malignancy. Histopathology showed no evidence of malignancy in the biopsy; however, a tissue culture showed growth of *Staphylococcus aureus*, which was sensitive to ciprofloxacin and clindamycin.

The patient then received inpatient treatment with antibiotics, including ofloxacin ear drops as well as oral clindamycin and ciprofloxacin. Furthermore, the patient received periodic follow-up with otorhinolaryngology. With the administration of antibiotics, the patient showed significant clinical improvement in terms of pain, facial weakness, and trismus. A post-treatment CT scan showed a marked favorable therapeutic response, including marked improvement of the previously noted right infratemporal and nasopharyngeal mass.

## Discussion

SBO is a potentially ominous condition that most commonly originates from a preexisting external ear infection, with *Pseudomonas aeruginosa* being the causative organism in more than 90% of cases [1-2]. Other organisms such as *Staphylococcus aureus*, *Staphylococcus epidermidis*, and *Klebsiella* have been reported as less common causes of SBO [1]. Furthermore, SBO originating from a middle ear infection is a very rare occurrence [3].

Patients with SBO can present with several clinical features, such as profound and persistent otalgia, purulent otorrhea, spiking fever, aural fullness, and relentless headaches [4]. Additionally, the inflammation that results from the infection can lead to cranial neuropathies, most commonly affecting the abducens nerve [5]. However, this inflammation can also involve other cranial nerves, such as the facial, glossopharyngeal, and vagus nerves [6].

Our patient was an elderly male who was known to have diabetes mellitus and presented with suppurative otitis media for one month, accompanied by headache, right-sided facial weakness, and trismus. According to a systemic review of 42 cases, these symptoms are very common in the initial presentation of SBO [7]. Middle-aged and elderly males with diabetes or immunocompromised status are most commonly predisposed to SBO [7]. Approximately 30% of those patients with SBO had long-lasting neurological sequelae despite being treated aggressively with antibiotics [7]. Furthermore, a 10% mortality rate was found for SBO within this population [7].

As mentioned previously, SBO is commonly reported in the literature as a sequela of otitis externa, and there are scarce reports concerning the atypical origins of SBO (e.g., otitis media) [3]. At the time of presentation, our patient had evidence of otitis media (rather than otitis externa), which was thought to be the source of his SBO.

Typically, SBO is diagnosed based on patient history, physical examination, and imaging. If SBO is suspected, it is vital to identify and diagnose it at an early stage to prevent any complications to the nervous system. In some cases, the use of biopsy and histopathology is warranted to rule out malignancy. With regard to blood tests, SBO typically causes elevations in inflammatory markers such as the erythrocyte sedimentation rate, C-reactive protein, and white blood cell count [8]. With regard to imaging, CT and MRI are generally useful in the early identification of the infection in terms of location and extension [9-10].

Inpatient antibiotics, typically in the form of long-term broad-spectrum antibiotics, are considered the mainstay of treatment for bacterial SBO [11]. The choice of antibiotic to be used for this purpose varies, but some of the most typically used antibiotics are third-generation cephalosporins, B-lactamase antibiotics, and quinolones, depending on the indication [1-12]. Early intervention is of utmost importance in SBO because it is associated with fewer neurological complications and reduced rates of mortality and morbidity [11-12]. Patients with diabetes or immunocompromised status, in particular, require strict glycemic control and continuous immune status monitoring in order to have a better chance at a successful resolution of the infection [11-12].

## Conclusions

SBO is a serious condition associated with high rates of morbidity and mortality. One cause of morbidity in SBO is the involvement of cranial nerves, such as the facial and trigeminal nerves. Reports concerning the atypical origins of SBO, such as otitis media, in the literature are scarce. In addition to patient history and physical examination, CT and MRI play major roles in the diagnosis and post-treatment monitoring of SBO. In most cases, the cornerstone of SBO treatment is antibiotics, which should be tailored to each patient.
